# Orbital Tuning of Tunnel Coupling in InAs/InP Nanowire Quantum Dots

**DOI:** 10.1021/acs.nanolett.9b04850

**Published:** 2020-02-12

**Authors:** Zahra Sadre Momtaz, Stefano Servino, Valeria Demontis, Valentina Zannier, Daniele Ercolani, Francesca Rossi, Francesco Rossella, Lucia Sorba, Fabio Beltram, Stefano Roddaro

**Affiliations:** † NEST, Instituto Nanoscienze CNR and Scuola Normale Superiore, Piazza S. Silvestro 12, I-56127 Pisa, Italy; ‡ Department of Physics “E.Fermi”, Università di Pisa, Largo Pontecorvo 3, I-56127 Pisa, Italy; § IMEM-CNR Institute, Parco Area delle Scienze, I-43124 Parma, Italy

**Keywords:** nanowire, quantum dot, InAs/InP, Coulomb blockade, tunnel barrier, electron tunneling rate

## Abstract

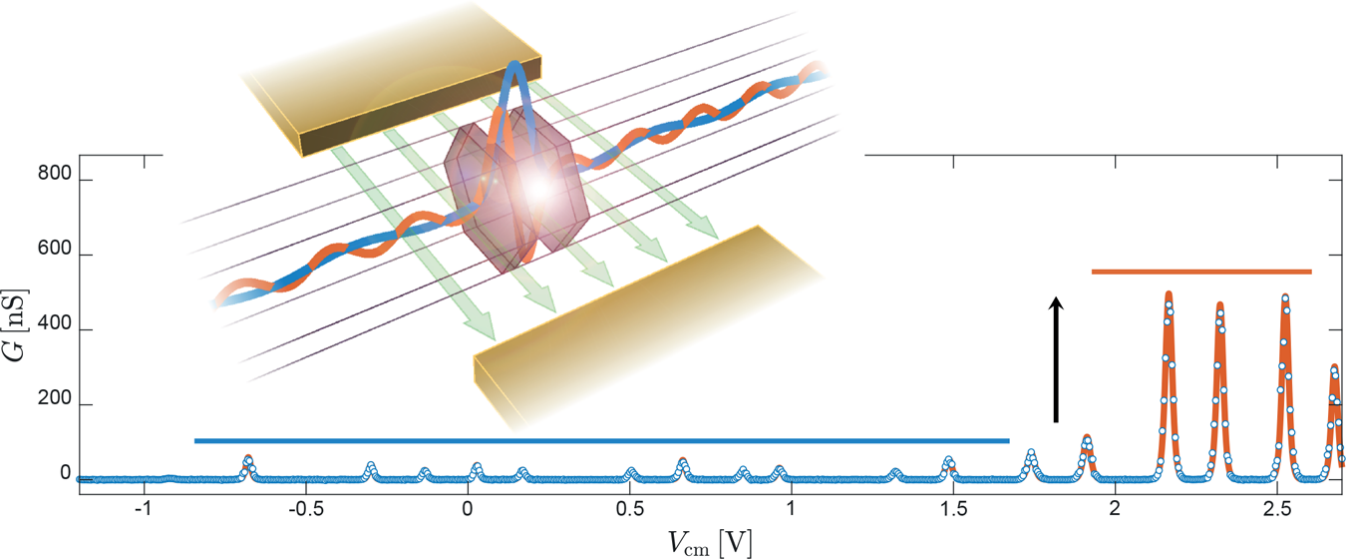

We report results on the control of barrier transparency in InAs/InP nanowire quantum dots via the electrostatic control of the device electron states. Recent works demonstrated that barrier transparency in this class of devices displays a general trend just depending on the total orbital energy of the trapped electrons. We show that a qualitatively different regime is observed at relatively low filling numbers, where tunneling rates are rather controlled by the axial configuration of the electron orbital. Transmission rates versus filling are further modified by acting on the radial configuration of the orbitals by means of electrostatic gating, and the barrier transparency for the various orbitals is found to evolve as expected from numerical simulations. The possibility to exploit this mechanism to achieve a controlled continuous tuning of the tunneling rate of an individual Coulomb blockade resonance is discussed.

Heterostructured InAs/InP nanowires (NWs) represent an ideal platform for the implementation of single-electron transistors^1−7^ and a variety of quantum devices for single-photon emission,^8^ spin manipulation,^5,9,10^ thermoelectric conversion,^11−13^ and more.^14^ These bottom-up nanostructures can indeed be grown with a fine control on the chemical composition along the NW axis and with atomically sharp interfaces;^1,15−17^ this, in combination with the small band mass and favorable Fermi pinning in InAs-based NWs, makes it possible to tightly confine electrons and yields nanostructures characterized by large confinement and charging energies.^2,5^ This growth control can be exploited to carefully tailor tunnel barrier properties but leads to tunnel couplings that are less obvious to tune with respect to the ones obtained in gated structures. This limits the successful exploitation of these nanostructures in many device architectures: tunnel coupling must be matched to the thermal energy scale for optimal thermoelectric conversion;^12,13,18^ single-photon detectors based on quantum dots (QDs) can require a wide range of different tunneling rates;^19^ the investigation of quantum and manybody phenomena such as those involving the Kondo effects^20−24^ and electron spectroscopy for the investigation of proximity effects^25−27^ typically requires the fine-tuning of high-quality tunnel barriers.

A strategy to achieve barrier-transparency tunability in these nanostructures can be based on the control of the orbital mediating conduction through the QD.^28,29^ In quantum well (QW) systems, tunnel coupling strongly depends on the subband index of the tunneling electrons but not on its in-plane dynamics, since the transverse momentum is conserved in the transmission process.^30^ In heterostructured NWs, electronic states are fully confined (both axial and radial directions) and a similar dependence may be expected as long as they can be roughly factored in a radial and axial part: the former would be conserved during tunneling through a clean barrier, and only the axial configuration of the orbital would matter. This is at odds with what is experimentally observed, and tunnel coupling was recently reported to display an overall trend consistent with a relatively simple monotonic dependence on the total orbital energy.^28^ Here, we show that tunnel rates in InAs/InP QDs can display a qualitatively different behavior in the low filling regime and that this is consistent with radial orbital-configuration conservation during tunneling. In our experiments, we use devices embedding a single InAs/InP QD and show that tunneling rates typically display a stepwise increase above a given filling threshold. We show that this is caused by the occupation of orbitals belonging to higher-index axial subbands that are naturally characterized by a larger barrier penetration. Our conclusions are crucially supported by the experimental configuration we adopted that allows us to separately control the radial confinement in the QD. This in turn allows us to alter the sequence of weakly/strongly coupled orbital states. As argued in the data discussion, our results indicate that a *continuous* tuning of the tunneling rate of individual Coulomb blockade peaks could be in principle achieved and controlled by field effect.

The structure of the devices used in the experiment is visible in Figure 1a. Fabrication started with the growth of heterostructured InAs/InP NWs by chemical beam epitaxy (CBE), using Au nanoparticles obtained by thermal dewetting of a thin Au film on InAs (111). The nanostructures have a nominal corner-to-corner “diameter” of 48 ± 5 nm and embed two 5 ± 1 nm InP barriers separated by a 19 ± 1 nm InAs island; see the scanning transmission electron micrograph in Figure 1b obtained using a high-angle annular dark field (HAADF) detector and depicting a NW nominally identical to the ones used to fabricate the devices. The broad scattering in the NW parameters is due to the size distribution of the Au nanoparticles obtained by thermal dewetting; significantly sharper distributions are obtained using other methods.^15,31,32^ In the scanning electron micrograph, the NW is deposited on top of a degenerately doped Si substrate covered by 300 nm of SiO_2_. Device fabrication is completed by thermal evaporation of two Ti/Au (10/100 nm) Ohmic contacts acting as the source (S) and drain (D) electrodes (blue in Figure 1a). In addition, two 200 nm-wide local side-gate electrodes named sg1 and sg2 (yellow) are aligned at the two sides of the NW, in correspondence to the InAs/InP heterostructure. Further details about device structure and measurement setup are reported in the Supporting Information.

**Figure 1:**
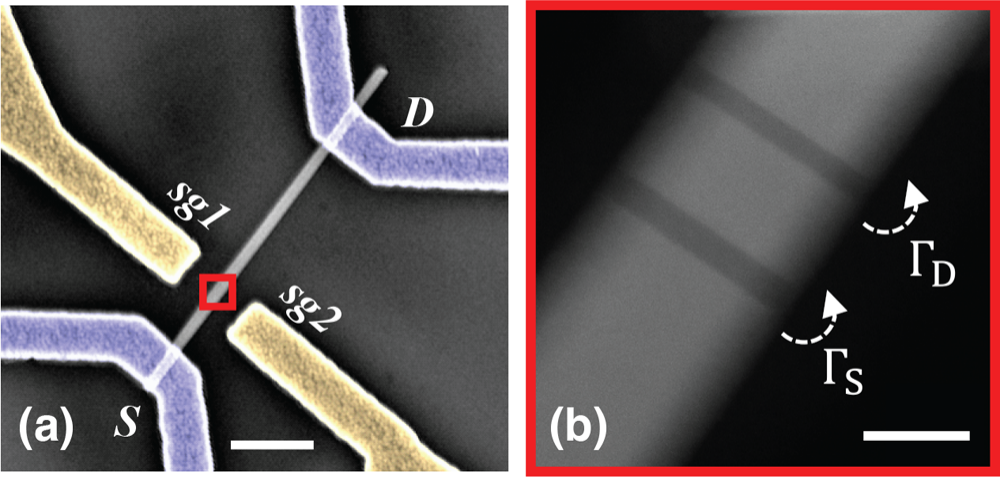
Device architecture. (a) The studied devices are fabricated on top of doped SiO_2_/Si and feature source and drain Ohmic contacts (blue) and two lateral field-effect gates (yellow). (b) *Z*-contrast STEM image of the core of the device, which is a heterostructured InAs/InP nanowire embedding two InP barriers defining a 19 ± 1 nm-thick InAs island. Scale bars in the two panels correspond to 400 and 20 nm, respectively.

Figure 2 reports experimental data and shows that the evolution of tunnel coupling is not trivially connected to the total orbital energy. The two lateral gates and the Si substrate can be used to control the number of electrons (*N*) in the QD and the local electrostatic environment. In particular, the substrate gate is used to enhance carrier density in the NW and to ensure that the source and drain sections of the device display a robust conductance; in the experiments reported in the paper, the substrate is held at a potential of +5.5 V. The lateral electrodes can control the value of *N* and the QD spectrum, depending on the specific bias configuration. In Figure 2a, side gates are biased at the same voltage *V*_cm_; i.e., they are operated in a “common mode”—and control the QD filling. The color plot reports the absolute current value in logarithmic scale as a function of *V*_cm_, starting from pinch-off (*N* = 0) up to a filling exceeding 20 electrons. A typical even–odd filling pattern is obtained, as expected from spin-degeneracy in the quantum Coulomb blockade regime. Charging energies and level spacing can be extracted from the height of odd and even diamonds. Further data analysis is reported in the Supporting Information and yields an average charging energy of *E*_C_ ≈ 5 meV, level spacings in the range Δε = 0–15 meV, and the common-mode lever arm α_cm_ = 0.04–0.08 meV/V. The observed energy scales are compatible with prior experimental reports^2,5,33,34^ with the exception of the side-gate lever arm that strongly depends on the specific electrode geometry used in this experiment. Differential conductance data d*I*/d*V* in Figure 2b highlight the presence of a clear threshold in the QD conductance when *N* > 14.

**Figure 2:**
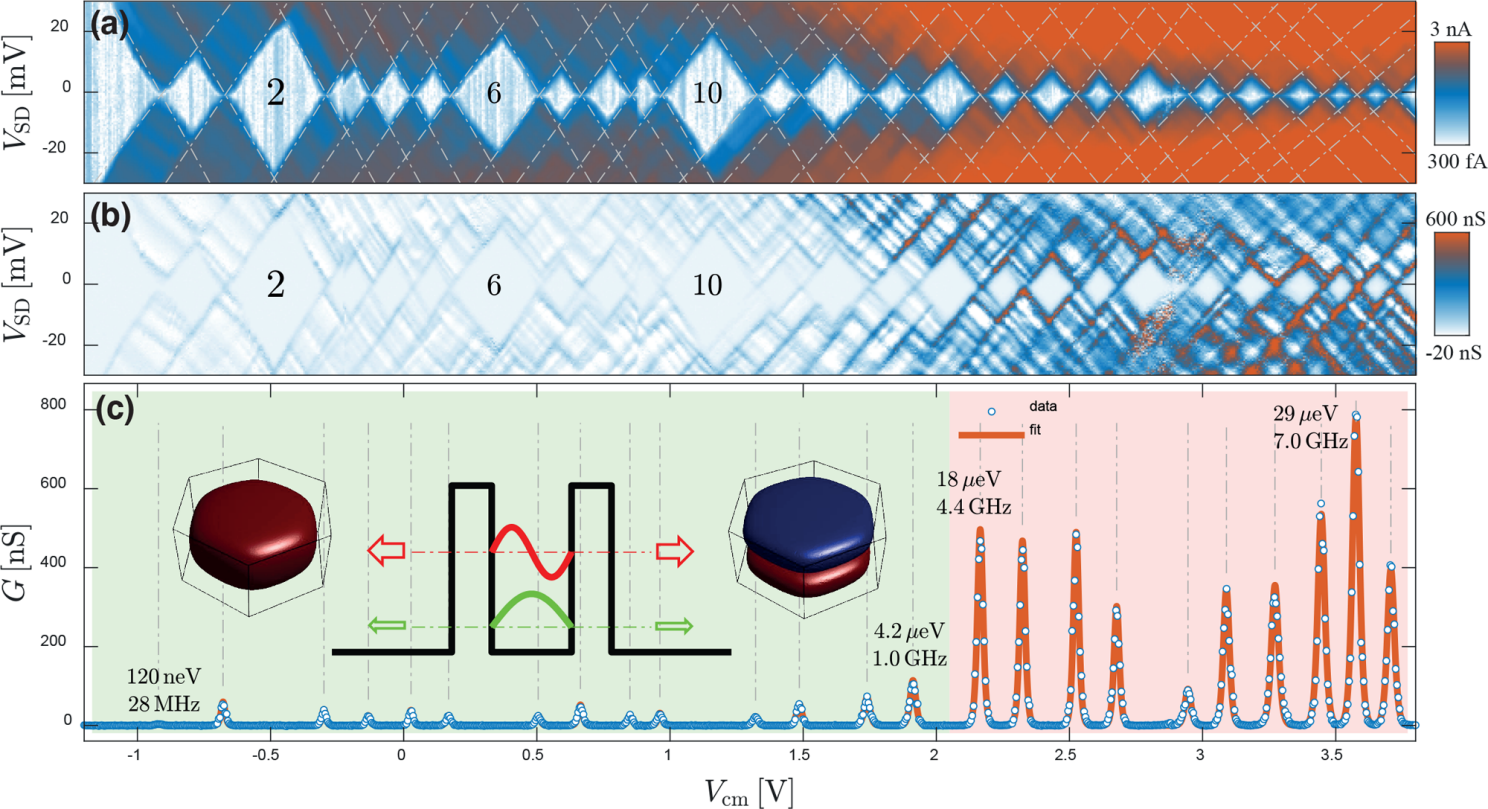
Orbital dependence of the tunnel coupling. (a) Coulomb blockade diagram as a function of the common-mode side gate voltage *V*_cm_ for one of the studied devices. The color plot displays the absolute current in logarithmic scale and covers a fairly wide range of fillings going from *N* = 0 up to beyond 20 electrons. (b) The equivalent plot of the differential conductance d*I*/d*V* in linear scale highlights the presence of a conductance threshold for *V*_cm_ ≈ 2 V at *N* = 14. (c) The zero-bias differential conductance *G* is fitted using standard single-level line shapes, yielding a good agreement with the data with a temperature of *T* = 4.14 K. As discussed in the main text, the presence of a conductance threshold can be understood in terms of the population of orbitals with different axial quantum numbers, as schematized in the overlay.

Figure 2c displays the corresponding zero-bias differential conductance *G* = d*I*/d*V*|_0_: we fit the data using the line shape expected for a nondegenerate delta-like resonance and estimate the total serial tunnel rate Γ = Γ_S_Γ_D_/(Γ_S_ + Γ_D_), where Γ_S/D_ are the source and drain barrier couplings.^35^ The tunnel amplitude is in this case simply linked to the peak amplitude, while the broadening is dominated by thermal effects. The studied devices display Coulomb peaks with Γ values that stochastically change from orbital to orbital but fall in the range of a few hundred neV (green). From *V*_cm_ ≈ 2.0 V, a transition to more strongly coupled resonances is observed (in the range of tens of μeV, red), with an abruptness depending on the specific device tested. The observed stepwise pattern of the tunneling rates is obviously inconsistent with a mere stochastic dependence of the tunneling on the individual orbitals; moreover, it is also not simply linked to the total electron energy, as previously observed for QDs with similar nominal dimensions operated at much larger electron filling values.^28^ In fact, here we focus on a complementary regime close to the QD pinch-off: as we shall argue in the following, our data indicate a different phenomenology for which the sudden increase in the tunnel coupling is driven by the occupation of orbitals belonging to the second axial subband in the region confined between the two InP barriers, as sketched in the overlay to Figure 2c. This interpretation is supported by an accurate experimental analysis of the QD filling and energy spectrum, also as a function of an adjustable radial confinement.

In order to discuss the observed evolution of the electron tunneling rates as a function of *N*, we report in Figure 3 the result of a simulation of the orbital states in the InAs/InP island. Orbitals are calculated using a single-particle approximation and assuming a hexagonal InAs box with an axial thickness of τ = 19.5 nm and a corner-to-corner “diameter” of *d* = 48 nm (see the sketch inset in the bottom left corner). If the radial confinement potential is large with respect to the energy scales of the problem, one can factor the wave function in terms of radial and axial components labeled by radial and axial quantum numbers *n*_r_ and *n*_a_ (see the Supporting Information). In particular, assuming a hard-wall potential, the total energy turns out to be equal to
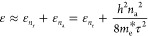
1where *n*_a_ is a positive integer and *m*_e_^*^ = 0.04*m*_e_ is the effective mass in wurzite InAs. In this ideal textbook limit, which turned useful in the interpretation of previous experiments,^10,36^ the complete QD spectrum is thus expected to contain many shifted copies of the radial excitation spectrum ε_*n*_r__, one for each value of the axial quantum number *n*_a_. This is clearly visible in the evolution of the spin-degenerate orbital energy ε(*E*_*x*_) as a function of an electric field *E*_*x*_ applied in the *x* direction in the radial plane of the QD (see Figure 3). For instance, the *n*_a_ = 1 and *n*_a_ = 2 states labeled as “A” have the same radial configuration and have a similar evolution as a function of *E*_*x*_; an equivalent correspondence is visible for each radial state (the ones labeled as “B” and so on). Importantly, as long as the conservation of transverse momentum holds, the tunnel rate depends only on *n*_a_: all of the *n*_a_ = 1 orbitals are expected to display the same tunnel coupling to the leads; *n*_a_ = 2 ones should exhibit a larger tunneling probability owing to their sizably larger ε_*n*_a__ and thus to their larger barrier penetration. We note that the density of states in the NW leads can in principle also play a role, but it is typically found to mostly give rise to mesoscopic fluctuations and is neglected in the current analysis. This observation provides a first rationalization of the observed device behavior: similar resonance amplitudes are observed as long as only *n*_a_ = 1 orbitals are populated, while the filling of those derived from the *n*_a_ = 2 subband leads to a larger conductivity through the QD. The experimentally observed threshold *N* ≈ 10–20 (see the Supporting Information) is consistent with the τ/*d* ≈ 0.5 ratio in our QDs. The electric field *E*_*x*_ breaks the symmetry of the radial confinement, lifts level degeneracy, and further confines electrons in the radial direction, thus modifying ε_*n*_r__. This leads to a general enhancement of the energy spacing between radial states in each ε_*n*_r__ sequence and thus to crossings between orbitals with different *n*_a_. As discussed in the following, this behavior is consistent with our experimental observations and indicates a possible mechanism yielding the controlled smooth tuning of electron tunneling rates in these nanostructures.

**Figure 3:**
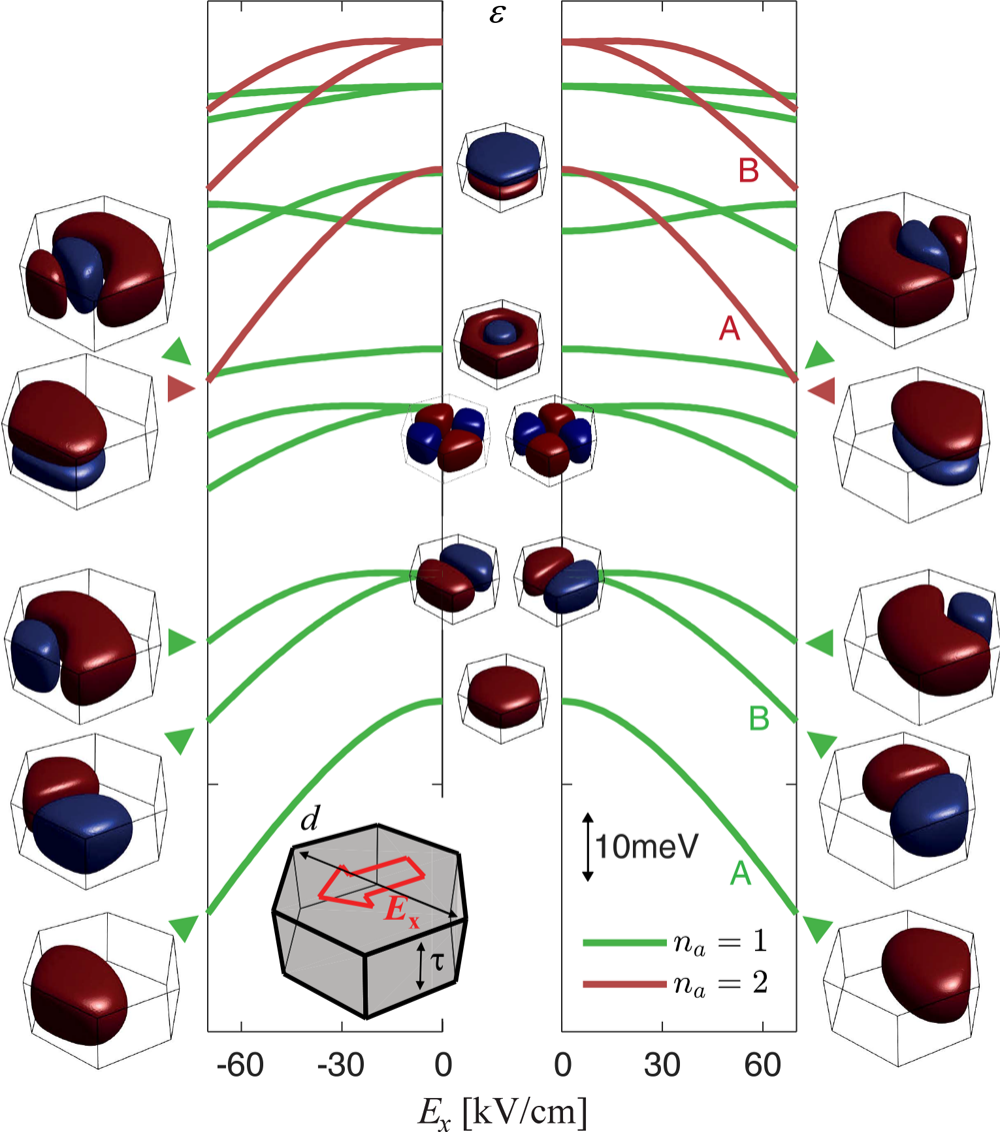
Orbital states in a hexagonal InAs island. Single-particle calculation of the orbital states in the InAs/InP QD as a function of an electric field applied along the *x* direction (see inset sketch). The confinement potential is approximated as a hard-wall hexagonal box with a “corner-to-corner” diameter of *d* = 48 nm and a thickness of τ = 19.5 nm. Lowest-laying orbitals display a decreasing energy at large *E*_*x*_ and are confined to the hexagon corner of lowest potential energy. All of the states marked in green correspond to wave functions originating from the radial confinement of the first axial subband; higher energy copies of the same spectral lines are visible in the red sequence and have two lobes in the axial direction. In this approximation, tunnel rates only depend on *n*_a_ and green orbitals are expected to display a smaller tunnel coupling with respect to the red ones.

In previous works, some of us demonstrated that the energy spectrum of InAs/InP QDs can be strongly modified using a local multiple-gating scheme.^4^ To a first approximation, the application of a differential bias between two gates sg1 and sg2 leads to the establishment of an electric field *E*_*x*_ in the QD region. As a result, the energy spectrum is modified. This is experimentally illustrated in Figure 4 where we report the evolution of the measured current as a function of the filling number and of the differential voltage between the two side gates. The results are presented as a color map of the QD current at a bias of 1 meV as a function of the common-mode voltage *V*_cm_ (controlling *N*) and of a differential voltage Δ*V* = *V*_sg1_ – *V*_sg2_ (controlling the transverse electric field *E*_*x*_). The difference Δ*V* was distributed on the two side gates according to the equations
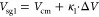
2
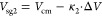
3where the κ parameters satisfy κ_1_ + κ_2_ = 1. The values of κ_1_ and κ_2_ are chosen so that the average *N* does not depend on Δ*V* and an approximately symmetric evolution is observed for positive and negative values of Δ*V*. In the specific case shown here, this leads to κ_1_ = 0.36 and κ_2_ = 0.64, with the asymmetry being due to a nonintentional nonsymmetric arrangement of the two local gates.

**Figure 4:**
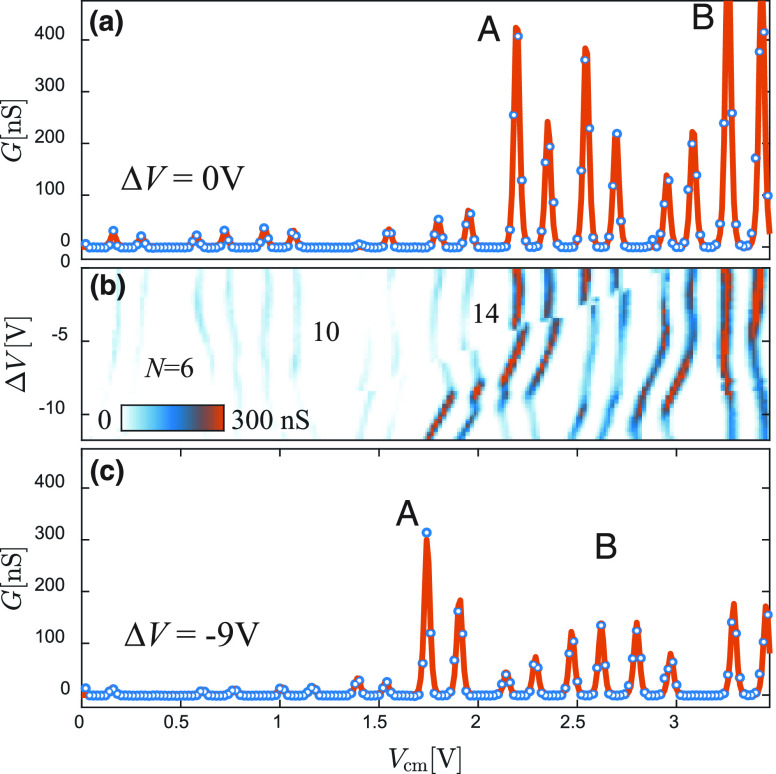
Spectral modulation and tunnel coupling. The application of a differential voltage Δ*V* to the side gates leads to a significant modulation of the Coulomb peak spacing and of the peak amplitudes. (a) At Δ*V* = 0 V, an onset to stronger Coulomb resonances is observed beyond *N* ≈ 14 and large conductivity peaks marked by a letter “A” are observed at *V*_cm_ ≈ 2.2 V. (b) The application of a finite Δ*V* leads to clear crossings between orbitals: in particular, the orbital labeled as “A” moves to lower energies and the onset to larger conductance peaks shifts to *N* = 12. A similar evolution is highlighted by the label “B”. (c) The cross section at Δ*V* = −9 V clearly shows the new position of the larger conductance peak and the occurrence of a strongly nonmonotonic evolution of Γ as a function of *N*.

The initial spectral configuration at Δ*V* = 0 is visible in Figure 4a, where the cross section as a function of *V*_cm_ matches the one of the data set in Figure 2c. As an increasing Δ*V* is applied to the side gates, Coulomb blockade peaks display a sizable evolution (Figure 4b) eventually leading to the configuration visible in Figure 4c when Δ*V* = −9*V*. We note that the threshold to the more strongly coupled orbitals shifts to lower values of *N*, consistently with the evolution in Figure 3. A first rough estimate of the electric field at a given Δ*V* can be made based on numerical simulations (see the Supporting Information), and for |Δ*V*| = 9 V, we expect *E*_*x*_ ≈ 45 kV/cm. This is in fact a consequence of an energy crossing between QD orbitals, which is clearly visible in the color plot of Figure 4b thanks to the strong orbital dependence of the amplitude of the Coulomb blockade peaks. In particular, the spin doublet A, visible at *V*_cm_ ≈ 2.2 V for zero detuning, clearly drifts to lower values of *V*_cm_, until it crosses another orbital at Δ*V* ≈ −9 V and is finally located at *V*_cm_ ≈ 1.8 V at the maximum explored value of Δ*V*. A further strongly coupled peak double B starts at *V*_cm_ ≈ 3.3 V and shifts to about 2.5 V. A similar evolution is observed for positive values of Δ*V*, as discussed later on.

The analysis of Figure 4 highlights a few important experimental facts. First of all, the increase in tunneling amplitude Γ is not simply monotonic in the orbital energy and thus in *N*. This emerges clearly by looking at the evolution of the Coulomb blockade as a function of Δ*V*: beyond a threshold, orbitals with different *n*_a_ can easily occur at energies that are too close to be resolved and/or are partially mixed, making it difficult to identify the nature of the QD orbitals involved. Differently, considering the overall spectral evolution of Figure 4b, specific orbitals with a stronger tunneling emerge and maintain their (larger) tunnel amplitude throughout the evolution versus Δ*V*. Peaks with larger tunneling rates tend to shift toward *lower* values of *N* for increasing values of Δ*V* (i.e., increasing transverse electric field *E*_*x*_). Such a behavior is consistent with the predictions of Figure 3 and can be understood as a consequence of the radial confinement of the states. We further note that the presence of level crossings between strongly and weakly coupled orbitals could—in the presence of a sufficiently large anticrossing, and thus hybridization, between the two wave functions—lead to a continuous tuning of the Coulomb blockade amplitude versus Δ*V*. In the current experiment, the two crossing levels are too close to be resolved so that a suitable anticrossing and hybridization should be introduced in the system.

The overall evolution of the QD spectrum in the filling range from *N* = 6 to *N* = 22 is illustrated in Figure 5a, extending the data set visible in Figure 4b. The good stability of the studied nano-heterostructures allows the investigation of a fairly large range of gate configurations, but charge rearrangements could not be completely avoided. Beyond the diagonal one visible in the color plot, a vertical rearrangement at Δ*V* ≈ 4 V was numerically removed by shifting the value of *V*_cm_ to improve readability (raw data are reported in the Supporting Information). Orbitals with larger tunneling are visible in red and clearly reproduce the crossing scheme reported in Figure 3, with an approximate mirror symmetry around the central dashed line. While at Δ*V* ≈ 0 the transition to larger tunneling resonances occurs after *N* = 14, at the configurations highlighted by the outer dashed lines, the transition shifts to *N* = 10–12. We stress that, even if only two QD orbitals in Figure 5a exhibit a larger tunneling amplitude, for generic Δ*V* values many more CB peaks display an amplified amplitude. This behavior is likely connected to a hybridization between strongly and weakly coupled orbitals and leads to intermediate (and not small) tunnel amplitudes. This highlights that a multigate architecture is needed to study the effect and identify the role of the involved orbitals in the QD. For selected values of Δ*V* (e.g., along the left dashed line), low-tunneling orbitals can be energetically decoupled and CB with a small amplitude can be observed even at relatively large *N* values, confirming our interpretation. We finally note that a precise matching between experimental data and Figure 3 cannot be expected owing to the approximations used: even restricting the analysis to the single-particle picture, band bending (inside the QD and at the NW surface) was not taken into account.

**Figure 5:**
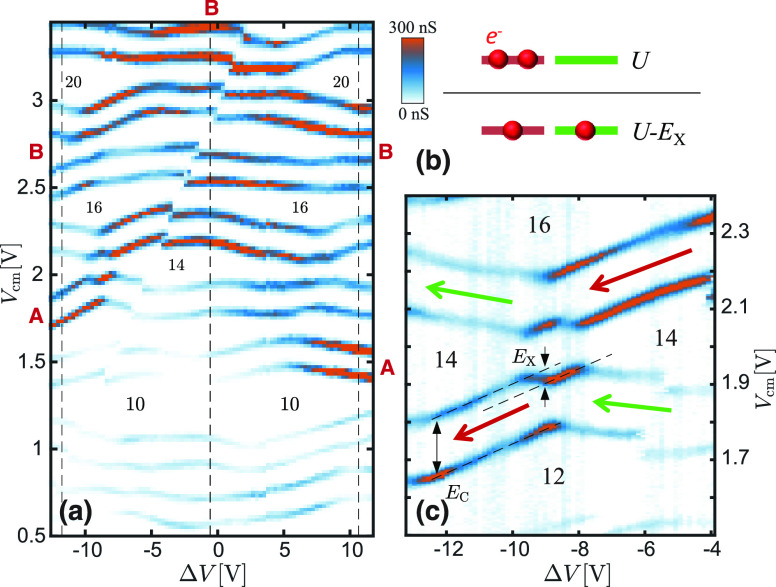
Overall spectral evolution of the QD. (a) Full evolution of the Coulomb blockade peaks versus the gate imbalance Δ*V* and the common-mode voltage *V*_cm_. Measurements were obtained using a bias of 1 mV and cover fillings from *N* = 6 to *N* = 22. Two resonances with a larger tunnel amplitude are highlighted by the letters “A” and “B”, consistently with Figure 3. (b) Higher-resolution current map in correspondence of one of the level crossings breaking the even–odd filling scheme. The phenomenology is known to derive from the reduction of the potential energy *U* of the last two electrons in the QD thanks to a partial filling of two nearly degenerate levels (see sketch on the top of the panel), for instance, as a consequence of exchange interaction. An energy gain of *E*_X_ = 1.85 ± 0.13 meV can be determined from the distance in *V*_cm_ between the dashed lines and from the QD lever arm at *N* ≈ 14.

Figure 5a shows that level crossing at times breaks the typical even–odd filling sequence and cannot be described by a standard constant interaction model: in Figure 5b, we report a finer map of the one at *N* = 14 for Δ*V* < 0. This class of crossings is found to occur particularly frequently when they are associated with a degeneracy between orbitals with different *n*_a_. The breakdown of the even–odd scheme was previously highlighted in the literature in association with level crossings induced by magnetic fields,^37^ local gating,^38^ or—similarly to the case reported here—radial confinement.^5,36^ The effect is fundamentally connected to the existence of a reduction of electrostatic cost *U* in the case of a partial occupation of two nearly degenerate orbitals, with respect to the more conventional complete filling of the lowest energy one (see sketch on the top of Figure 5b). In our case, we estimate a reduction of *E*_X_ = 1.85 ± 0.13 meV based on the *V*_cm_ shift between the dashed line and on the lever arm α_cm_ = 0.050 ± 0.0035 eV/V. The origin of the effect is typically related to exchange interaction,^37^ but recent works on similar NW systems have convincingly demonstrated that a major role can also be played by the spatial segregation of the two orbitals, leading to the formation of an effective parallel double QD system with a sizable reduction of direct Coulomb repulsion for the partial filling configuration.^6,7^ The main difference between the two scenarios is the degeneracy of the manybody configuration. In the exchange scenario, it is reduced by the lifting between *S* = 1 triplet and *S* = 0 singlet states, while a full spin degeneracy is expected when the energy gain of the partial filling of the two nearly degenerate orbitals has a purely Coulombian origin. While the effect represents an intriguing aspect of the phenomenology of the nanodevices, our current NWs do not provide a definite answer: on one hand, devices do not display sufficiently clear excited state patterns to establish the presence of a singlet–triplet splitting (further evidence available in the Supporting Information); on the other one, the studied QD structure does not present obvious features leading to the formation of a parallel double dot at the crossing between states with different *n*_a_. We note, however, that the orbital with a larger *n*_a_ will be naturally more confined in the radial direction with respect to the one with lower *n*_a_, possibly explaining—within the spatial segregation scenario—the occurrence of the effect.

In conclusion, we have demonstrated that the evolution of tunneling rates in InAs/InP QDs at small filling numbers displays a filling threshold leading to resonances with larger amplitude. The effect is understood in terms of the occupation of orbital states originating from different axial subbands confined between the two InP barriers and characterized by a larger tunneling probability. Our conclusion critically depends on the possibility to modulate the QD spectrum and reorder the sequence of orbitals with larger and smaller tunneling amplitudes, leading to a clear identification of the role of the different orbitals. Our work indicates an effective route to continuous and controllable tuning of the tunnel coupling in heterostructured NW systems.

## Methods

InAs/InP heterostructured NWs were grown by chemical beam epitaxy seeded by metallic nanoparticles obtained from thermal dewetting of a Au thin film. Growth was performed at 390 ± 10 °C using trimethylindium (TMIn: 0.3 Torr, cracked at the NW surface), *tert*-butylarsine (TBA: 1.0 Torr cracked at 1000 °C), and tributylphosphine (TBP: 4.0 Torr, cracked at 1000 °C). NWs have a wurtzite crystal structure; InAs/InP and InP/InAs interfaces were realized without any interruption by switching group-V precursors. The average position of the QD along the NW and from Au nanoparticles is 500 ± 20 nm, which was estimated based on transmission electron microscopy (JEOL JEM 2200 FS operated at 200 kV), leading to a typical alignment error of ±50 nm in fabrication. Ohmic contacts were obtained by thermal evaporation of a Ti/Au (10/100 nm) bilayer, after a chemical passivation step using a (NH_4_)S_*x*_ solution.^39^ The orbital configurations in the QD system were simulated using a commercial PDE solver (COMSOL Multiphysics). Transport measurements were performed in a Heliox system running at 4.2 K, using Yokogawa and Stanford Research System voltage sources and a low-noise DL-1211 current preamplifier.

## Supporting Information Available

Parameters of the device reported in the main text, numerical models, raw version of the color map in Figure 5, finite bias and breakdown of the even–odd filling, and device structure and measurement setup (PDF)

## References

[ref1] Björk M.; Ohlsson B.; Sass T.; Persson A.; Thelander C.; Magnusson M.; Deppert K.; Wallenberg L.; Samuelson L. One-dimensional steeplechase for electrons realized. Nano Lett. 2002, 2, 87–89. 10.1021/nl010099n.

[ref2] Björk M. T.; Thelander C.; Hansen A. E.; Jensen L. E.; Larsson M. W.; Wallenberg L. R.; Samuelson L. Few-electron quantum dots in nanowires. Nano Lett. 2004, 4, 1621–1625. 10.1021/nl049230s.

[ref3] Fuhrer A.; Fröberg L. E.; Pedersen J. N.; Larsson M. W.; Wacker A.; Pistol M.-E.; Samuelson L. Few electron double quantum dots in InAs/InP nanowire heterostructures. Nano Lett. 2007, 7, 243–246. 10.1021/nl061913f. 17297985

[ref4] Roddaro S.; Pescaglini A.; Ercolani D.; Sorba L.; Beltram F. Manipulation of electron orbitals in hard-wall InAs/InP nanowire quantum dots. Nano Lett. 2011, 11, 1695–1699. 10.1021/nl200209m. 21446718

[ref5] Romeo L.; Roddaro S.; Pitanti A.; Ercolani D.; Sorba L.; Beltram F. Electrostatic spin control in InAs/InP nanowire quantum dots. Nano Lett. 2012, 12, 4490–4494. 10.1021/nl301497j. 22849393

[ref6] Nilsson M.; Boström F. V.; Lehmann S.; Dick K. A.; Leijnse M.; Thelander C. Tuning the two-electron hybridization and spin states in parallel-coupled InAs quantum dots. Phys. Rev. Lett. 2018, 121, 156802 10.1103/PhysRevLett.121.156802. 30362807

[ref7] Nilsson M.; Chen I.-J.; Lehmann S.; Maulerova V.; Dick K. A.; Thelander C. Parallel-coupled quantum dots in InAs nanowires. Nano Lett. 2017, 17, 7847–7852. 10.1021/acs.nanolett.7b04090. 29172541

[ref8] Dalacu D.; Mnaymneh K.; Lapointe J.; Wu X.; Poole P. J.; Bulgarini G.; Zwiller V.; Reimer M. E. Ultraclean emission from InAsP quantum dots in defect-free wurtzite InP nanowires. Nano Lett. 2012, 12, 5919–5923. 10.1021/nl303327h. 23066839

[ref9] Björk M.; Fuhrer A.; Hansen A.; Larsson M.; Fröberg L.; Samuelson L. Tunable effective g factor in InAs nanowire quantum dots. Phys. Rev. B: Condens. Matter Mater. Phys. 2005, 72, 201307 10.1103/PhysRevB.72.201307.

[ref10] Rossella F.; Bertoni A.; Ercolani D.; Rontani M.; Sorba L.; Beltram F.; Roddaro S. Nanoscale spin rectifiers controlled by the Stark effect. Nat. Nanotechnol. 2014, 9, 997 10.1038/nnano.2014.251. 25383514

[ref11] Matthews J.; Hoffmann E. A.; Weber C.; Wacker A.; Linke H. Heat flow in InAs/InP heterostructure nanowires. Phys. Rev. B: Condens. Matter Mater. Phys. 2012, 86, 174302 10.1103/PhysRevB.86.174302.

[ref12] Josefsson M.; Svilans A.; Burke A. M.; Hoffmann E. A.; Fahlvik S.; Thelander C.; Leijnse M.; Linke H. A quantum-dot heat engine operating close to the thermodynamic efficiency limits. Nat. Nanotechnol. 2018, 13, 920 10.1038/s41565-018-0200-5. 30013221

[ref13] Prete D.; Erdman P. A.; Demontis V.; Zannier V.; Ercolani D.; Sorba L.; Beltram F.; Rossella F.; Taddei F.; Roddaro S. Thermoelectric Conversion at 30 K in InAs/InP Nanowire Quantum Dots. Nano Lett. 2019, 19, 3033–3039. 10.1021/acs.nanolett.9b00276. 30935206

[ref14] Nylund G.; Storm K.; Lehmann S.; Capasso F.; Samuelson L. Designed Quasi-1D Potential Structures Realized in Compositionally Graded InAs1–x P x Nanowires. Nano Lett. 2016, 16, 1017–1021. 10.1021/acs.nanolett.5b04067. 26788886

[ref15] Zannier V.; Rossi F.; Ercolani D.; Sorba L. Growth dynamics of InAs/InP nanowire heterostructures by Au-assisted chemical beam epitaxy. Nanotechnology 2019, 30, 094003 10.1088/1361-6528/aaf7ab. 30537697

[ref16] Zannier V.; Rossi F.; Dubrovskii V. G.; Ercolani D.; Battiato S.; Sorba L. Nanoparticle stability in axial InAs–InP nanowire heterostructures with atomically sharp interfaces. Nano Lett. 2018, 18, 167–174. 10.1021/acs.nanolett.7b03742. 29186660

[ref17] Ercolani D.; Rossi F.; Li A.; Roddaro S.; Grillo V.; Salviati G.; Beltram F.; Sorba L. InAs/InSb nanowire heterostructures grown by chemical beam epitaxy. Nanotechnology 2009, 20, 505605 10.1088/0957-4484/20/50/505605. 19907063

[ref18] Nakpathomkun N.; Xu H. Q.; Linke H. Thermoelectric efficiency at maximum power in low-dimensional systems. Phys. Rev. B: Condens. Matter Mater. Phys. 2010, 82, 235428 10.1103/PhysRevB.82.235428.

[ref19] Kleinschmidt P.; Giblin S.; Tzalenchuk A.; Hashiba H.; Antonov V.; Komiyama S. Sensitive detector for a passive terahertz imager. J. Appl. Phys. 2006, 99, 114504 10.1063/1.2199107.

[ref20] Goldhaber-Gordon D.; Shtrikman H.; Mahalu D.; Abusch-Magder D.; Meirav U.; Kastner M. Kondo effect in a single-electron transistor. Nature 1998, 391, 156 10.1038/34373.

[ref21] Cronenwett S. M.; Oosterkamp T. H.; Kouwenhoven L. P. A tunable Kondo effect in quantum dots. Science 1998, 281, 540–544. 10.1126/science.281.5376.540. 9677192

[ref22] Schmid J.; Weis J.; Eberl K.; Klitzing K. v. Absence of odd-even parity behavior for Kondo resonances in quantum dots. Phys. Rev. Lett. 2000, 84, 5824 10.1103/PhysRevLett.84.5824. 10991064

[ref23] Sasaki S.; De Franceschi S.; Elzerman J.; Van der Wiel W.; Eto M.; Tarucha S.; Kouwenhoven L. Kondo effect in an integer-spin quantum dot. Nature 2000, 405, 764 10.1038/35015509. 10866190

[ref24] Van der Wiel W.; De Franceschi S.; Fujisawa T.; Elzerman J.; Tarucha S.; Kouwenhoven L. The Kondo effect in the unitary limit. Science 2000, 289, 2105–2108. 10.1126/science.289.5487.2105. 11000108

[ref25] Jünger C.; Baumgartner A.; Delagrange R.; Chevallier D.; Lehmann S.; Nilsson M.; Dick K. A.; Thelander C.; Schönenberger C. Spectroscopy of the superconducting proximity effect in nanowires using integrated quantum dots. Communications Physics 2019, 2, 76 10.1038/s42005-019-0162-4.

[ref26] Doh Y.-J.; van Dam J. A.; Roest A. L.; Bakkers E. P.; Kouwenhoven L. P.; De Franceschi S. Tunable supercurrent through semiconductor nanowires. Science 2005, 309, 272–275. 10.1126/science.1113523. 16002611

[ref27] Deng M.; Vaitiekėnas S.; Hansen E. B.; Danon J.; Leijnse M.; Flensberg K.; Nygård J.; Krogstrup P.; Marcus C. M. Majorana bound state in a coupled quantum-dot hybrid-nanowire system. Science 2016, 354, 1557–1562. 10.1126/science.aaf3961. 28008065

[ref28] Thomas F. S.; Baumgartner A.; Gubser L.; Jünger C.; Fülöp G.; Nilsson M.; Rossi F.; Zannier V.; Sorba L.; Schönenberger C. Highly symmetric and tunable tunnel couplings in InAs/InP nanowire heterostructure quantum dots. Nanotechnology 2020, 31, 135003 10.1088/1361-6528/ab5ce6. 31778992

[ref29] Barker D.; Lehmann S.; Namazi L.; Nilsson M.; Thelander C.; Dick K. A.; Maisi V. F. Individually addressable double quantum dots formed with nanowire polytypes and identified by epitaxial markers. Appl. Phys. Lett. 2019, 114, 183502 10.1063/1.5089275.

[ref30] Luryi S. Frequency limit of double-barrier resonant-tunneling oscillators. Appl. Phys. Lett. 1985, 47, 490–492. 10.1063/1.96102.

[ref31] Messing M. E.; Hillerich K.; Bolinsson J.; Storm K.; Johansson J.; Dick K. A.; Deppert K. A comparative study of the effect of gold seed particle preparation method on nanowire growth. Nano Res. 2010, 3, 506–519. 10.1007/s12274-010-0011-y.

[ref32] Gomes U.; Ercolani D.; Zannier V.; Beltram F.; Sorba L. Controlling the diameter distribution and density of InAs nanowires grown by Au-assisted methods. Semicond. Sci. Technol. 2015, 30, 115012 10.1088/0268-1242/30/11/115012.

[ref33] Johnson A. C.; Petta J. R.; Marcus C.; Hanson M.; Gossard A. Singlet-triplet spin blockade and charge sensing in a few-electron double quantum dot. Phys. Rev. B: Condens. Matter Mater. Phys. 2005, 72, 165308 10.1103/PhysRevB.72.165308.

[ref34] Salfi J.; Roddaro S.; Ercolani D.; Sorba L.; Savelyev I.; Blumin M.; Ruda H.; Beltram F. Electronic properties of quantum dot systems realized in semiconductor nanowires. Semicond. Sci. Technol. 2010, 25, 024007 10.1088/0268-1242/25/2/024007.

[ref35] Ihn T. Semiconductor Nanostructures: Quantum states and electronic transport; Oxford University Press: 2010.

[ref36] Rossella F.; Ercolani D.; Sorba L.; Beltram F.; Roddaro S. Electrostatic spin control in multi-barrier nanowires. J. Phys. D: Appl. Phys. 2014, 47, 394015 10.1088/0022-3727/47/39/394015.

[ref37] Tarucha S.; Austing D.; Tokura Y.; Van der Wiel W.; Kouwenhoven L. P. Direct Coulomb and exchange interaction in artificial atoms. Phys. Rev. Lett. 2000, 84, 2485 10.1103/PhysRevLett.84.2485. 11018916

[ref38] Fuhrer A.; Ihn T.; Ensslin K.; Wegscheider W.; Bichler M. Singlet-triplet transition tuned by asymmetric gate voltages in a quantum ring. Phys. Rev. Lett. 2003, 91, 206802 10.1103/PhysRevLett.91.206802. 14683385

[ref39] Suyatin D.; Thelander C.; Björk M.; Maximov I.; Samuelson L. Sulfur passivation for ohmic contact formation to InAs nanowires. Nanotechnology 2007, 18, 105307 10.1088/0957-4484/18/10/105307.

