# Supplementary material for: Orbital Tuning of Tunnel Coupling in InAs/InP Nanowire Quantum Dots
Source: Nano Lett. 2020 Feb 12;20(3):1693–9. doi: 10.1021/acs.nanolett.9b04850 (PMC7997631; doi:10.1021/acs.nanolett.9b04850)
Supplement: Supplementary file 1 — nl9b04850_si_001.pdf [file nl9b04850_si_001.pdf]

# Supporting Information: Orbital Tuning of Tunnel Coupling in InAs/InP Nanowire Quantum dots

Zahra Sadre Momtaz,[*,†,§] Stefano Servino,[‡] Valeria Demontis,[†] Valentina Zannier,[†] Daniele Ercolani,[†] Francesca Rossi,[¶] Francesco Rossella,[†] Lucia Sorba,[†] Fabio Beltram,[†] and Stefano Roddaro[*,†,‡]

†*NEST, Instituto Nanoscienze CNR and Scuola Normale Superiore, Piazza S. Silvestro 12, I-56127 Pisa, Italy*

‡*Department of Physics "E.Fermi", Università di Pisa, Largo Pontecorvo 3, I-56127 Pisa, Italy*

¶*IMEM-CNR Institute, Parco Area delle Scienze, Parma, Italy*

§*Current address: Institut Néel, CNRS, 25av. des Martyrs, 38000 Grenoble, France*

E-mail: zahra.sadre-momtaz@neel.cnrs.fr; stefano.roddaro@unipi.it

## S.1 Parameters of the device reported in the main text

Three different devices were fully characterized within this study. The first one, whose results are presented in the main text, will be called hereafter D#1, and the other two devices will be called D#2 and D#3. Figure S1, reports the key experimental parameters of each of the measured devices. Experimental data was fitted using a constant interaction model and the resulting parameters are reported as a function of the filling number N, for every Coulomb blockade (CB) diamond. In particular, we report: (a) the addition energy ΔU;

(b) the common-mode gate lever arm, $\alpha_{cm}$; (c) the common-mode capacitance $C_{cm}$ and the total capacitance $C_\Sigma$ of the quantum dot. The parameters were calculated starting from a fit of the slopes of the Coulomb diamond's edges of the stability diagram. The ΔU values for odd diamonds (blue dots in the graph) correspond to the charging energies ($E_c$), while the energy difference between even (grey dots in the graph) and odd diamonds can be used to estimate the level spacing ($\Delta\varepsilon$). The charging energy in the QD of D#1 is about 10 meV, while the level spacing reaches the values up to 10-15 meV. The common-mode lever arm used for the energy conversion in Figure 5 of the main paper was obtained by a linear fit of the data reported above (around N=14).

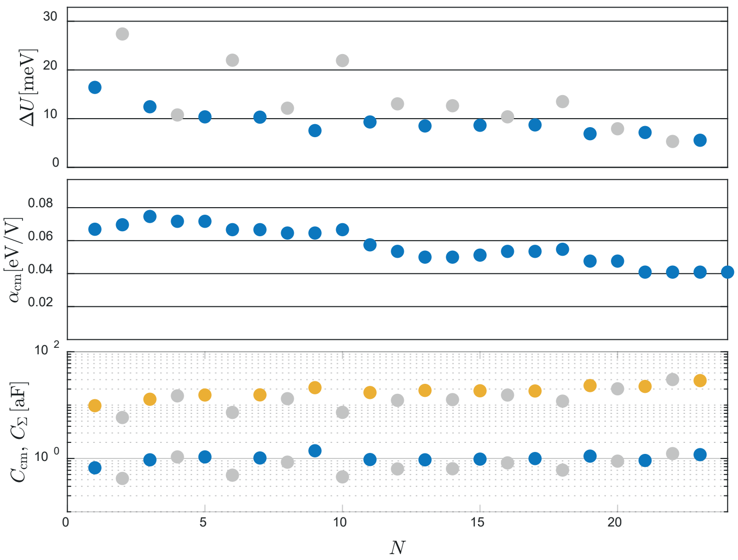


Figure S1: Main fit parameters of device D#1 (device reported in the main text).

The tunneling rate Γ for each peak was extracted by fitting the zero bias conductance peaks (Figure 2.c in the main text) using the typical line-shape for a non-degenerate delta-like resonances[1]

$$G = \frac{e^2}{h} \cdot \frac{\hbar\Gamma}{k_B T} \cdot \frac{1}{4\cosh^2[\alpha_k(V_g^N - V_g)/2k_B T]} \tag{1}$$

where $\alpha_k$ is the gate lever arm, $V_g$ is the gate voltage and $V_g^N$ is peak gate voltage at filling N. Corrections can be expected considering the spin degeneracy, but these effects were beyond the scope of the current paper. The results of the described fitting procedure are reported in the following table.

| $N$ | 1 | 2 | 3 | 4 | 5 | 6 | 7 | 8 | 9 | 10 | 11 | 12 |
|---|---|---|---|---|---|---|---|---|---|---|---|---|
| $\Gamma(\mu eV)$ | 0.17 | 2.2 | 1.5 | 0.95 | 1.4 | 0.96 | 0.93 | 1.9 | 0.98 | 1.2 | 0.86 | 2.0 |
| $\Gamma(GHz)$ | 0.04 | 0.54 | 0.36 | 0.23 | 0.35 | 0.23 | 0.23 | 0.47 | 0.24 | 0.28 | 0.21 | 0.49 |
| $N$ | 13 | 14 | 15 | 16 | 17 | 18 | 19 | 20 | 21 | 22 | 23 | 24 |
| $\Gamma(\mu eV)$ | 2.6 | 4.2 | 18 | 17 | 18 | 11 | 3.4 | 13 | 13 | 20 | 29 | 15 |
| $\Gamma(GHz)$ | 0.63 | 1.0 | 4.4 | 4.2 | 4.4 | 2.7 | 0.83 | 3.1 | 3.2 | 4.8 | 7.0 | 3.5 |

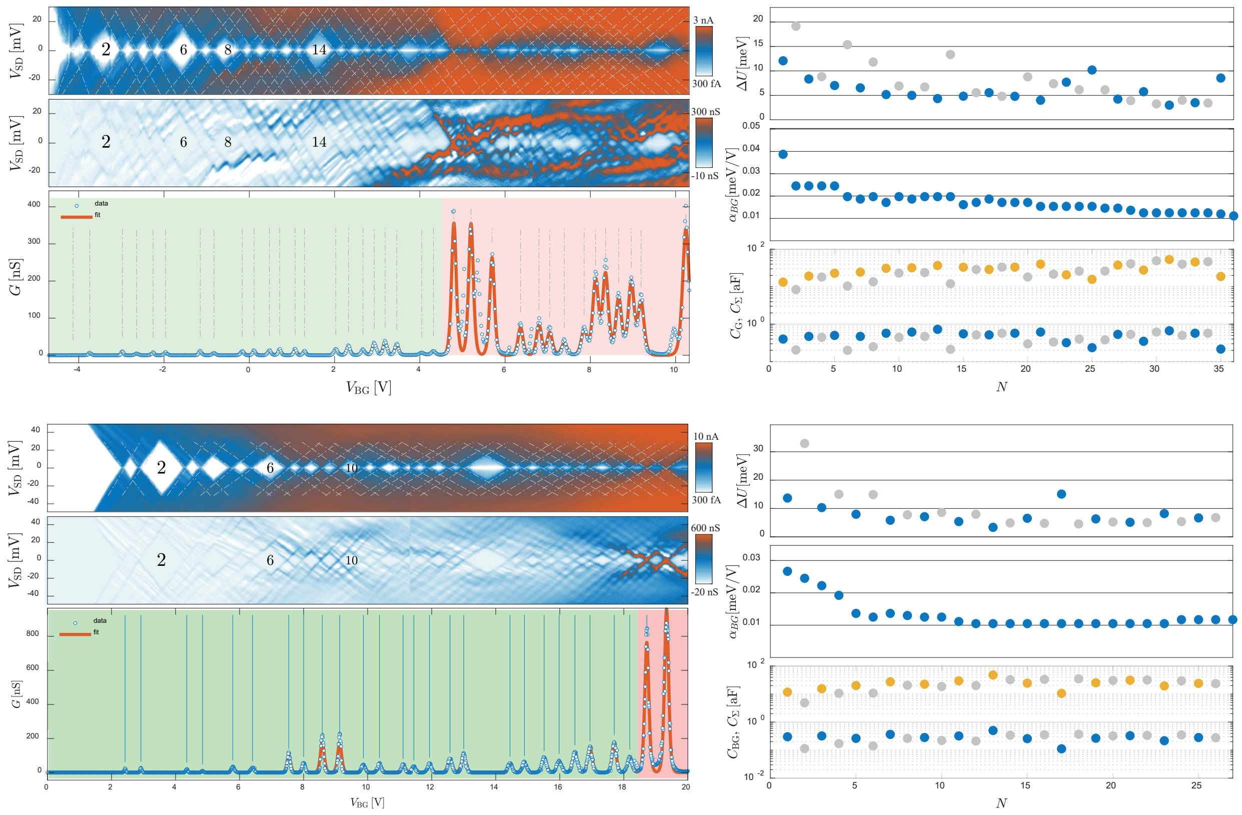


Figure S2. Left: Coulomb blockade diagrams for D#2 and D#3 as a function of the back gate voltage, absolute current in log scale and differential conductance at the bottom the zero-bias conductance is reported. Right: parameters extracted from the dataset. Note that the even-odd assignment is not reliable for large filling values due to the presence of charge rearrangements.

In Figure S2 we report the stability diagram and extracted parameters for D#2 and D#3. In the plot, the back gate electrode was used to control the electron population inside the QD and an abrupt threshold to larger tunneling was observed. Devices D#2 and D#3 displayed a similar tunability using lateral gates but a full investigation of the spectral dependence could not be performed due to the presence of too many charge rearrangements over the large voltage swings necessary to perform the study. The threshold for the observation of large coupling orbitals was observed to be $N = 22$ and $N \approx 24$ for devices D#2 and D#3, respectively. Note the electron filling in D#3 cannot be reliably identified due to the presence of a non-negligible charge rearrangement.

## S.2 Numerical models

The main target of the simulations was to estimate the expected spacing between radial and axial excitations in the studied QD, i.e. the number of orbitals that need to be filled before larger tunneling resonances can be experimentally observed. To this end, we made a set of simplified assumptions and used a single particle model to predict the expected energy spacings. The nominal geometry of the studied QDs is reported in Figure S3; the InAs island is a hexagonal box with an axial thickness $\tau = 19.5$ nm in the $z$ direction and a corner-to-corner diameter $d = 48$ nm.

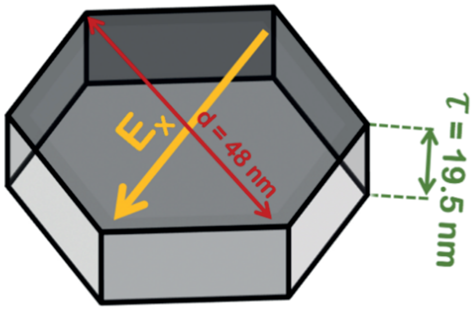

Figure S3: Sketch of the hexagonal box used as a model for the hard-wall NW QD

Calculations were performed using the PDE solver Comsol Multiphysics and the following further approximations were made: (i) the confinement potential is infinite and barrier penetration is neglected; (ii) band bending at the NW surface and the non-parabolicity effects are neglected. In these approximations, the wave function can be factored as the product

$\psi(x, y, z) = A(z)B(x, y)$, where the axial component has a particularly simple expression $A(z) = sin(kz)$ and $k_z = \pi n_a/\tau$ where $n_a$ is an integer. This leads to eigenvalues of the form

$$\varepsilon = \varepsilon_{n_r} + \varepsilon_{n_a} = \varepsilon_{n_r}(E_x) + \frac{h^2 n_a^2}{8 m_e^* \tau^2} \qquad (2)$$

where $n_r$ is the quantum number of the radial problem and the transvers electric field acts only on $\varepsilon_{n_r}$ . While the adopted approximations are clearly strong, the resulting estimates on the filling numbers were found to be reliable in the limit of low occupation numbers. Importantly, it has to be noted that in such a limit the transmission across the barrier only depends to the $z$ part of the eigenvalue problem, and thus only on $n_a$. In particular, larger transmission amplitudes can be expected for larger $k_z$ values and thus for larger $n_a$ quantum numbers. This is in full agreement with the experimental observations reported in the main text in Figure 2c.

In order to allow a semi-quantitative comparison between the single-particle orbital energies reported in the main text with the applied gate voltages, a set of 2D electrostatic simulations have been performed. Figure S4 shows the results for the transverse component of the electric field $E_x$ for the following gating configuration: $V_{cm} = 0V$, $\Delta V$ = 8V and $V_{BG}$ = 5.5 V. Known dielectric constants BG for silicon oxide and InAs were used to estimate the local electric field for a gate gap of 250 nm (based on SEM imaging of the measured device, see Figure S7). In the stated configuration, we obtain an average field in the $x$ direction

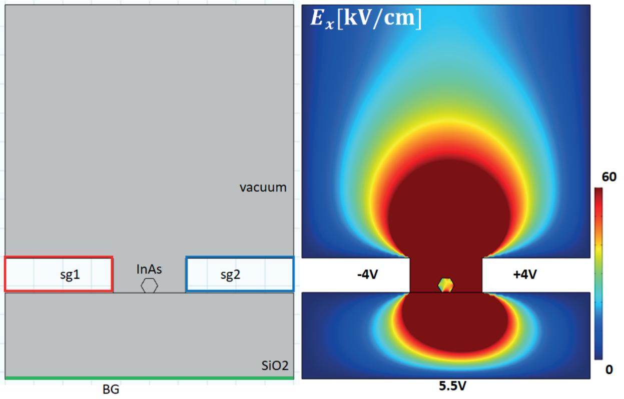

Figure S4: Finite element simulation of the electrostatics of the multi-gate architecture used for this study.

equal to about 40 kV/cm which leads to a consistent comparison between the energy plots reported in Figure 3 and those of Figure 5. The numerical result provides a first rough estimate of the correspondence between lateral gate imbalance and transverse fields: about 1 kV/cm can be expected for every $\approx 0.2$ V of imbalance between the lateral gates. We note the calculation does not take into account the (unknown) screening from mobile charges at the surface and in the bulk of the InAs nanowire and thus should be only regarded as a rough consistency check.

## S.3 Raw version of the colormap in Figure 5

Figure 5 in the main text shows the evolution of the Coulomb blockade peaks versus the gate imbalance $\Delta V$ and the common mode voltage $V_{cm}$ . The map required significant voltage swings on the two gates, up to voltages beyond $\pm 10V$. Despite the general very good stability of the studied devices, charge rearrangements could not be completely avoided over such large gate swings and imperfections occurred even in the most stable devices. In order to improve the readability of the plot a reproducible charge rearrangement occurring at $\Delta V \approx 4V$ was numerically removed in the color-plot reported in the main text. The original dataset can be seen on the right hand side on Figure S5.

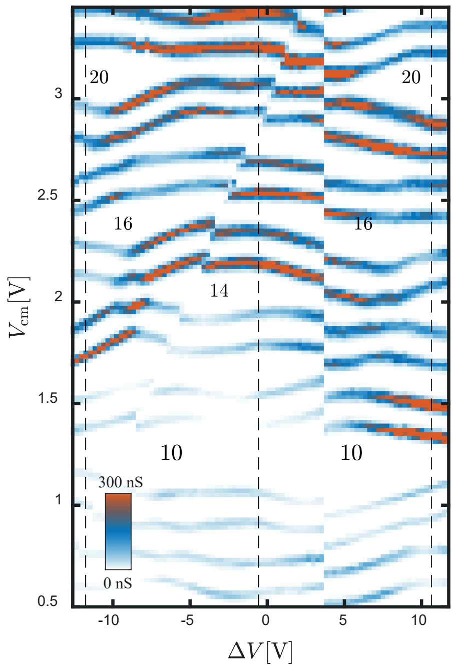


Figure S5: Raw data used for the plot visible in Figure 5 of the main text.

Filling number are determined according the correspondence with data in Figure 2.

## S.4 Finite bias data and breakdown of the even-odd filling

The physical origin of the breakdown of the even-odd filling sequence observed in the spectral evolution of Figure 5a, e.g. at the level crossing highlighted in Figure 5b, could not be conclusively identified in this work. Similar phenomenology has been reported in the literature and has been typically connected the role of exchange interaction in promoting a partial filling of two orbitals and a breakdown of the even-odd filling scheme. Another possibility is that direct Coulomb interaction between the two partially occupied orbitals is simply weaker, because electrons are spatially located in two different regions of the real space. This has been convincingly demonstrated in,[2] where two independent parallel dot are formed in the low filling regime, and it has also been suggested in,[3] where the presence of multiple barriers can be expected to lead to a natural localization of the orbitals between different pairs of consecutive barriers. The expected origin of the effect is less obvious to identify in the current device, but one experimental possibility consists in studying the excitation spectrum and look for evidences of a splitting between triplet and single states linked to the different possible spin configurations of the two partially occupied orbitals. The current devices do not provide clear evidence of the presence of such a splitting and no conclusive result could be reached.

In Figure S6 we report a sequence of diamond scans (differential conductance) as a function of the common-mode gate voltage $V_{cm}$, for a discrete set of imbalance values $\Delta V$ covering the "anomalous" crossing occurring at N=14 for $\Delta V \approx -9V$. Few excited state lines are visible in measurement but they appear to be connect with the partial or total filling of the available orbitals. For instance, in the sequence starting from imbalance -7V we highlighted an excitation line for the tunneling of the $14^{th}$ electron. The excitation energy is found to decrease until the size of the N=14 diamond is minimized at imbalance -8.12 V. This voltage marks the beginning of the gating region where the even-odd filling scheme is violated. This excitation line is obviously related to a partial filling of both the crossing orbitals and the process becomes the lowest energy one after imbalance -8.12 V. In the case

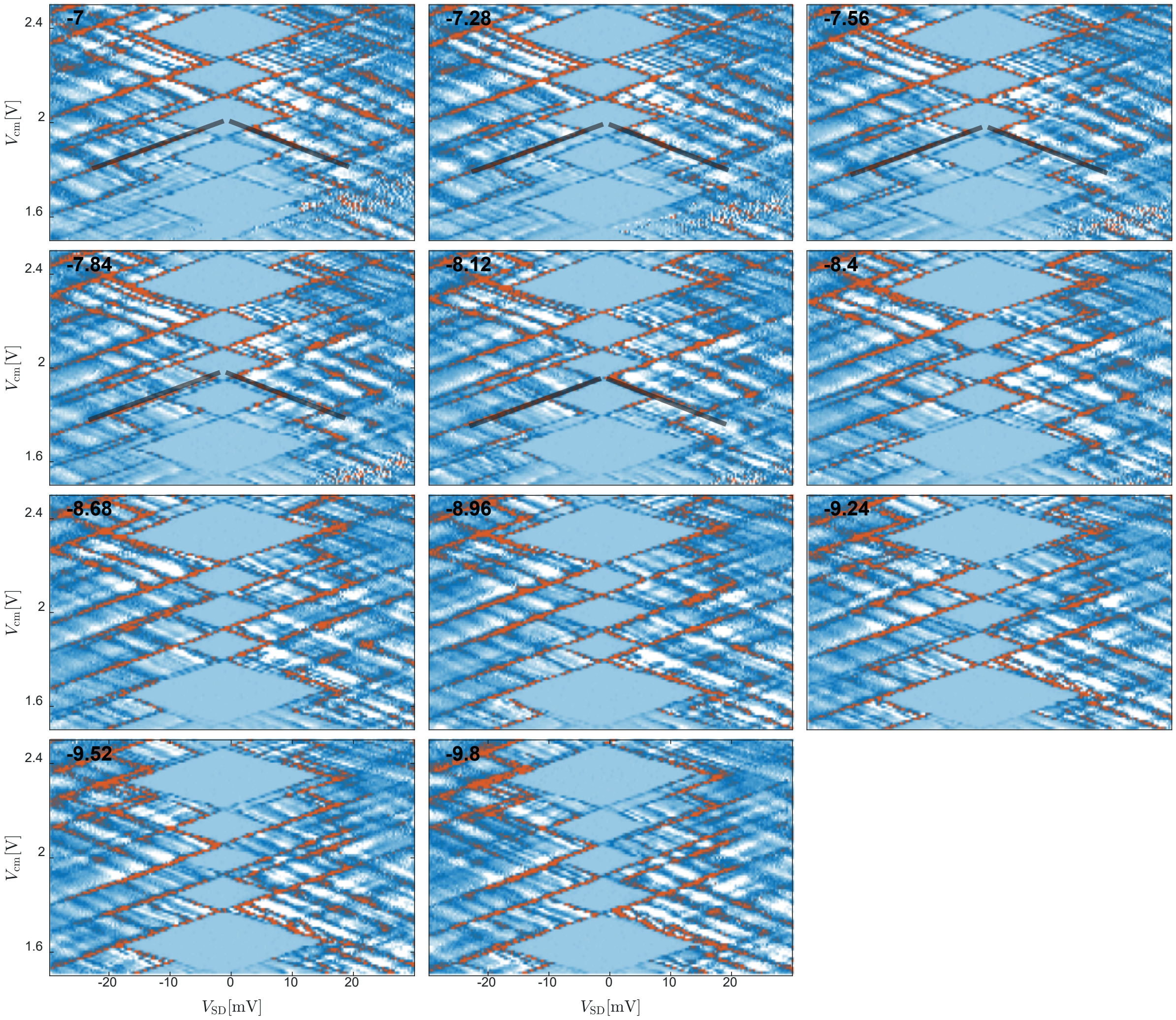


Figure S6: Diamond scans obtained at different cross-sections at fixed detuning $\Delta V$ in the filling range going from N=12 (bottom diamond) up to N=16 (top diamond)

of exchange interaction, one would expect the presence of both a S=1 (lower energy) and S=0 (higher energy) resonance for such a process. According to the energy gain of 1.86 meV for the partial filling of both orbitals, in the case of exchange the singlet-triplet gap would be expected to be about 3.5 meV.[4] No obvious evidence for such a further resonance could be highlighted.

## S.5 Device structure and measurement set-up

The structure of the studied devices is shown in Figure S7 along with a sketch of the measurement setup. Different gaps between the gates (GG) have been studied in different devices. The device analyzed in the main text is reported in the SEM picture and had a gate gap of about 250 nm.

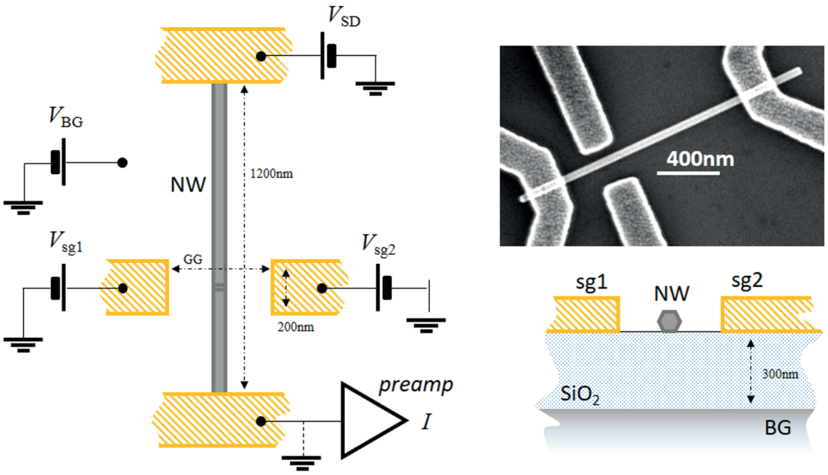


Figure S7: Device structure, SEM picture of the device studied in the main text, and sketch of the measurement setup.

## References


(1) Beenakker, C. Theory of Coulomb-blockade oscillations in the conductance of a quantum dot. *Physical Review B* **1991**, *44*, 1646.

(2) Nilsson, M.; Boström, F. V.; Lehmann, S.; Dick, K. A.; Leijnse, M.; Thelander, C. Tuning the two-electron hybridization and spin states in parallel-coupled InAs quantum dots. *Physical review letters* **2018**, *121*, 156802.

(3) Rossella, F.; Ercolani, D.; Sorba, L.; Beltram, F.; Roddaro, S. Electrostatic spin control in multi-barrier nanowires. *Journal of Physics D: Applied Physics* **2014**, *47*, 394015.

(4) Tarucha, S.; Austing, D.; Tokura, Y.; Van der Wiel, W.; Kouwenhoven, L. P. Direct Coulomb and exchange interaction in artificial atoms. *Physical review letters* **2000**, *84*, 2485.
